# The cost of primary care consultations associated with long COVID in non-hospitalised adults: a retrospective cohort study using UK primary care data

**DOI:** 10.1186/s12875-023-02196-1

**Published:** 2023-11-20

**Authors:** Jake Tufts, Naijie Guan, Dawit T Zemedikun, Anuradhaa Subramanian, Krishna Gokhale, Puja Myles, Tim Williams, Tom Marshall, Melanie Calvert, Karen Matthews, Krishnarajah Nirantharakumar, Louise J Jackson, Shamil Haroon

**Affiliations:** 1 University Hospitals of Morecambe Bay NHS Foundation Trust, Lancashire, LA9 7RG UK; 2 Institute of Applied Health Research, University of Birmingham, Edgbaston, Birmingham, B15 2TT UK; 3 School of Population and Global Health (M431), The University of Western Australia, 35 Stirling Highway, Perth, WA 6009 Australia; 4 Clinical Practice Research Datalink, Medicines and Healthcare Products Regulatory Agency, London, E14 4PU UK; 5 Birmingham Health Partners Centre for Regulatory Science and Innovation, University of Birmingham, Birmingham, B15 2TT UK; 6 Applied Research Collaboration (ARC) West Midlands, National Institute for Health Research (NIHR), Birmingham, CV4 7AJ UK; 7 NIHR Birmingham Biomedical Research Centre, University Hospital Birmingham and University of Birmingham, Birmingham, B15 2TH UK; 8 NIHR Birmingham-Oxford Blood and Transplant Research Unit (BTRU) in Precision Transplant and Cellular Therapeutics, University of Birmingham, Birmingham, B15 2TT UK; 9 Long Covid SOS, Charity Registered in England & Wales, 11A Westland Road, Faringdon, SN7 7EX Oxfordshire UK

**Keywords:** COVID-19, Long COVID, Economic, Cost, Primary care

## Abstract

**Background:**

The economic impact of managing long COVID in primary care is unknown. We estimated the costs of primary care consultations associated with long COVID and explored the relationship between risk factors and costs.

**Methods:**

Data were obtained on non-hospitalised adults from the Clinical Practice Research Datalink Aurum primary care database. We used propensity score matching with an incremental cost method to estimate additional primary care consultation costs associated with long COVID (12 weeks after COVID-19) at an individual and UK national level. We applied multivariable regression models to estimate the association between risk factors and consultations costs beyond 12 weeks from acute COVID-19.

**Results:**

Based on an analysis of 472,173 patients with COVID-19 and 472,173 unexposed individuals, the annual incremental cost of primary care consultations associated with long COVID was £2.44 per patient and £23,382,452 at the national level. Among patients with COVID-19, a long COVID diagnosis and reporting of longer-term symptoms were associated with a 43% and 44% increase in primary care consultation costs respectively, compared to patients without long COVID symptoms. Older age, female sex, obesity, being from a white ethnic group, comorbidities and prior consultation frequency were all associated with increased primary care consultation costs.

**Conclusions:**

The costs of primary care consultations associated with long COVID in non-hospitalised adults are substantial. Costs are significantly higher among those diagnosed with long COVID, those with long COVID symptoms, older adults, females, and those with obesity and comorbidities.

**Supplementary Information:**

The online version contains supplementary material available at 10.1186/s12875-023-02196-1.

## Introduction

Long COVID is one of the largest public health challenges associated with the COVID-19 pandemic. The World Health Organisation defines it as the continuation or development of new symptoms three months from probable or confirmed Severe Acute Respiratory Syndrome Coronavirus-2 (SARS-CoV-2) infection, with symptoms lasting for at least two months, that cannot be explained by an alternative diagnosis [1, 2]. The prevalence of long COVID in the UK and worldwide is high [3]. In June 2022, two million people were estimated to be experiencing self-reported long COVID in the UK alone [3]. At the time of the current study, over 630 million people worldwide had cumulatively had COVID-19 [4] and 6.2% were estimated to have experienced symptoms lasting beyond three months from infection [5], suggesting a global long COVID prevalence of approximately 40 million cases. This burden has steadily increased over the course of the pandemic and of those self-reporting long COVID, 72% reported that their symptoms were adversely affecting their day-to-day activities [6].

Research has shown that in comparison to uninfected individuals, those with a history of COVID-19, the vast majority of whom were not hospitalised, had significantly higher GP consultation rates post-infection [7, 8]. It is therefore likely that long COVID has also led to increased primary care costs but no robust evidence on this has currently been published. Estimating the cost of primary care consultations attributed to long COVID can help inform understanding of the economic burden of the condition on health services. Analysing how the costs vary across population subgroups and how they are influenced by risk factors can inform healthcare policy and decisions relating to resource allocation.

The aim of the study was to estimate the excess primary care costs associated with consultations to support non-hospitalised people with long COVID. The three objectives were to estimate the incremental costs of these consultations per patient with a history of COVID-19 beyond 12 weeks from infection, to estimate the national primary care costs of these consultations in the UK, and to assess the association between demographic and clinical risk factors with incremental costs among those with a history of COVID-19. Our study aimed to estimate the cost of long COVID from a primary care perspective, by quantifying the direct healthcare costs from primary care consultations that can be attributed to supporting people with long COVID, compared to a closely matched cohort of individuals with no record of suspected or confirmed COVID-19 [9].

## Methods

### Study design

A retrospective matched cohort study was conducted using data from a large primary care database based in the UK. The study compared the frequency and costs of primary care consultations in a cohort of individuals with confirmed SARS-CoV-2 infection, at least 12 weeks after infection (representing the longer-term effects of COVID-19 or post-COVID-19 condition/long COVID), to a propensity score matched cohort of individuals without suspected or confirmed COVID-19 (Appendix S1). The costs associated with additional primary care consultations to support those with long COVID were estimated for the UK. Healthcare resource use was calculated using a bottom-up approach, and incremental costs were estimated using the matched control method [10, 11]. The association between patient characteristics and primary care consultation costs among those with confirmed SARS-CoV-2 infection were then assessed. This analysis was part of the Therapies for Long COVID in non-hospitalised individuals (TLC) Study [12].

### Data source

Data were obtained from the Clinical Practice Research Datalink (CPRD) Aurum database from 31^st^ January 2020 to 15^th^ April 2021 [13]. CPRD Aurum contains anonymised routinely collected data from UK general practices that use the EMIS Web® patient record system [14]. In June 2021, over 13 million actively registered patients were included in CPRD Aurum, covering approximately 20% of the UK population and 15% of all general practices in the UK [13]. The database is representative of the UK population and captures data on patient demographics, diagnoses, symptoms and more. SNOMED CT terms were used for coding diagnoses and symptoms [12, 15]. Data extraction was performed using the Data Extraction for Epidemiological Research (DExtER) tool for automated clinical epidemiological studies [16].

### Study population

Patients were sampled from general practices that were eligible if they had provided research quality data for at least 12 months before the study start date (31^st^ January 2020). Patients were eligible if they were 18 years or older, had been registered with a general practice for more than 12 months, and had a minimum of 12 weeks of follow-up. The latter eligibility criterion was included as long COVID is defined as symptoms persisting beyond 12 weeks of infection so a minimum of 12 weeks of follow-up was needed to assess resource use beyond this period. Patients were excluded if they transferred out of their practice during the study period for any reason other than death. This was done to capture the full history of resource use and expenditure.

Two cohorts of patients were sampled. The exposed cohort were adults with a SARS-CoV-2 infection confirmed by a reverse transcriptase polymerase chain reaction (RT-PCR) or lateral flow antigen test (see Supplementary Table 1 for SNOMED-CT codes) and had not been hospitalised 14 days before or 42 days after infection (within 28 days of infection with a ± 14-day grace period for clinical coding delays) [17]. Long COVID is underdiagnosed and poorly coded in primary care records and hence coded diagnoses of long COVID were not used to define the exposed cohort [18]. The unexposed cohort consisted of propensity score-matched (Appendix S1 and Supplementary Table 2) adults with no record of a positive RT-PCR or lateral flow antigen test for SARS-CoV-2, and no documented diagnoses of suspected or confirmed COVID-19 during the study period, and had not been hospitalised during a matched time period. These individuals were allocated a matched index date to account for immortal time bias [19] using the Data Extraction for Epidemiological Studies (DExTER) platform [16]. Within the exposed cohort, two subgroups were defined as those with a coded diagnosis of long COVID (DLC) and those reporting at least one of the recognised symptoms in the WHO diagnostic criteria for long COVID (SLC), 12 weeks after initial infection (Supplementary Tables 3 and 4).

### Follow-up

The follow-up period was defined as the time between a patient’s index date (date of SARS-CoV-2 infection in the exposed cohort or matched time point in the unexposed cohort) and the patient’s study end date. This was defined as the earliest of the following time points: study end date (15^th^ April 2021), death date, or the last date of data collection from the practice. Supplementary Fig. 1 depicts a timeline showing the study dates and time periods of interest.

### Outcomes and costing method

The primary outcome was the occurrence of a primary care consultation, defined as either a general practitioner (GP), nurse, or physiotherapy appointment at least 12 weeks after the index date (also see Supplementary Tables 5 and 6). Costs of consultations were estimated with unit costs for healthcare resources being taken from the Personal Social Services Research Unit’s (PSSRU) Unit Costs of Health and Social Care 2021, to represent the cost perspective of the UK National Health Service (NHS) [20]. The hourly cost for each healthcare professional and the average consultation duration were used to calculate consultation costs. Multiple consultations on the same day with the same healthcare professional were counted as a single consultation [21]. Details of the cost estimation are provided in Supplementary Tables 7, 8 and 9 and Appendix S2.

### Statistical analysis

First, the difference between the matched groups in total costs for primary care consultations was calculated within the matched follow-up period. Bootstrapped t-tests and analysis of variance (ANOVA) were used to compare means across the exposed and unexposed cohorts and the predefined subgroups. A multivariable ordinary least squares (OLS) regression model was also used to assess the incremental cost while adjusting for relevant confounding factors. The proportion of consultation costs associated with each professional group (GP, nurse, and physiotherapist) and consultation type (telephone, in-person appointment, home visit, and triage) was also calculated.

Then, cumulative COVID-19 incidence estimates from the UK Office for National Statistics (ONS) in the COVID-19 Infection Survey, were used to estimate the national incremental costs attributed to primary care consultations for non-hospitalised patients with long COVID across the whole UK population [22]. Details are provided in Appendix S2.

We used a log OLS regression model to explore the cost predictors of primary care consultations in patients with a history of COVID-19, where the dependent variable was transformed by the natural logarithm. The model included the DLC and SLC subgroups as covariates and adjusted for the same covariates used for the propensity score model (Supplementary Table 2).

A sensitivity analysis was then conducted to assess the assumption that follow-up time does not confound the costs. It included patients with at least six months of follow-up time from their index date, focusing on cost data from three to six months from the index date.

Missing data was denoted by a missing category within the variable. All statistical analyses were performed using STATA version 17 and R version 4.2.0.

## Results

### Study population

There were 472,173 patients in both the exposed and unexposed cohorts (Supplementary Fig. 2). The diagnosed long COVID (DLC) and symptomatic long COVID (SLC) subgroups consisted of 3,871 (0.8%) and 30,174 (6.4%) patients, respectively. The matched groups were similar in each of the baseline characteristics including age, sex, ethnic group, socioeconomic status, smoking status, body mass index (BMI), the number of prior consultations, and a wide range of comorbidities (Table 1, Supplementary Table 10, and Supplementary Figs. 3 and 4). The mean age was 44 years, 55% were female, and 64% belonged to a white ethnic group. 22% were current smokers and just over 55% were overweight or obese. The basic characteristics of individuals in our exposed cohort are similar to those reported by the UK Health Security Agency, where the mean age of the COVID-19 infected people in England was approximately 41 years, and 55% were females [23].

**Table 1:** Baseline characteristics of the matched exposed and unexposed groups

Variables	Unexposed (*n* = 472,173), n (%)	Exposed (*n* = 472,173), n (%)
Age (mean (SD))	44.14 (16.92)	44.16 (16.86)
Sex		
Male	210,848 (44.7)	211,683 (44.8)
Female	261,325 (55.3)	260,490 (55.2)
Ethnicity		
White	300,873 (63.7)	299,609 (63.5)
Asian	59,720 (12.6)	60,544 (12.8)
Black	18,572 (3.9)	18,598 (3.9)
Mixed	9,410 (2.0)	9,448 (2.0)
Other	7,266 (1.5)	7,208 (1.5)
Missing	76,332 (16.2)	76,766 (16.3)
IMD		
1 (Least deprived)	74,314 (15.7)	74,123 (15.7)
2	76,964 (16.3)	76,637 (16.2)
3	80,474 (17.0)	80,293 (17.0)
4	96,097 (20.4)	96,506 (20.4)
5 (Most deprived)	102,825 (21.8)	103,331 (21.9)
Missing	41,499 (8.8)	41,283 (8.7)
Smoking Status		
Current smoker	104,696 (22.2)	104,986 (22.2)
Ex-Smoker	165,128 (35.0)	163,759 (34.7)
Never smoked	159,371 (33.8)	159,655 (33.8)
Missing	42,978 (9.1)	43,773 (9.3)
BMI category		
Normal weight	143,346 (30.4)	144,426 (30.6)
Underweight	17,363 (3.7)	17,483 (3.7)
Obese	127,060 (26.9)	125,086 (26.5)
Overweight	139,589 (29.6)	138,694 (29.4)
Missing	44,815 (9.5)	46,484 (9.8)
Number of consultations 3 to 12 months prior to index date (mean (SD))		
GP	2.12 (3.68)	2.15 (3.44)
Nurse	0.50 (1.59)	0.50 (1.49)
Physiotherapist	0.01 (0.19)	0.01 (0.19)
Surgery	1.22 (2.24)	1.20 (2.03)
Home visits	0.03 (0.45)	0.07 (0.79)
Telephone	1.37 (2.73)	1.38 (2.52)
Triage	0.02 (0.30)	0.02 (0.27)

*n* The number of patients in that category, *%* Percentage of the group in the category, *SD* Standard deviation

### Incremental costs

Table 2 shows the number of consultations and associated costs for patients 12 weeks from their index date between 15^th^ April 2020 and 15^th^ April 2021. The numbers of primary care consultations were 209,620 (0.44 per patient) in the unexposed cohort and 245,177 (0.54 per patient) in the exposed cohort, respectively. The exposed cohort had a 22.7% higher relative rate of consultations, compared to patients in the unexposed cohort.

**Table 2:** Estimates of the annual primary care resource use and costs associated with Long COVID between 15^th^ April 2020 and 15^th^ April 2021

**Cost component**	**Main analysis cohorts:**		**COVID-19 patients with:**	
	**Unexposed (*n* = 472,173)**	**Exposed (*n* = 472,173)**	**DLC**^**1**^** (*n* = 3,871)**	**SLC**^**2**^** (*n* = 30,174)**
**Consultations 12 weeks after index date**				
Count	209,620	254,177	6,156	83,202
Rate (per patient)	0.44	0.54^***^	1.59^***^	2.76^***^
**Cost (absolute)**				
Total	£5,384,140	£6,533,404	£162,289	£2,080,873
Per patient	£11.40	£13.84^***^	£41.92^***^	£68.96^***^
**Cost (per person year)**				
Total	£11,284,942	£13,856,145	£363,739.70	£3,915,701
Mean	£23.90	£29.35^***^	£93.97^***^	£129.77^***^

Unexposed cohort: a pool of the eligible patients without a record of COVID-19. Exposed cohort: non-hospitalised patients with confirmed SARS-CoV-2 infection. *DLC* Diagnosed with Long COVID patient group, *SLC* Symptoms of Long COVID patient group

*Key:* ^*^p < 0.05, ^**^p < 0.01, ^***^p < 0.001

The incremental cost of primary care consultations beyond 12 weeks from infection for the exposed cohort compared to the unexposed cohort was £2.44 per patient per year. Using OLS regression, the coefficient for belonging to the exposed cohort is interpreted as a £2.09 cost increase per exposed patient, supporting the main analysis (Supplementary Tables 11 and 12). DLC and SLC subgroups’ consultation rates were over 3 and 6 times greater, with incremental costs of £30.52 and £57.56 per patient, respectively.

**Fig. 1:**
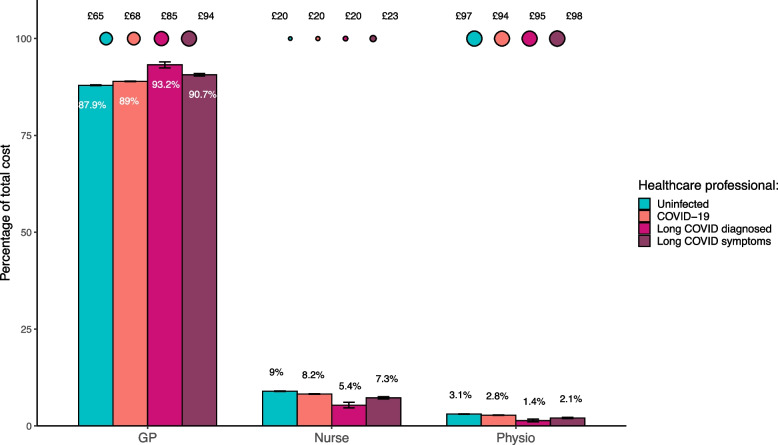
Bubble plot showing the average cost of each healthcare professional per patient (who had a consultation) between 15^th^ April 2020 and 15^th^ April 2021. Bar chart to show the percentage makeup of each group’s total costs by healthcare professional

GP consultations were the largest contributor to total costs for each exposure group, representing over 85% of costs (Fig. 1), and made up proportionately more of the total cost for the exposed and DLC and SLC subgroups than the unexposed cohort (*p* < 0.01). The average cost per patient was higher for all COVID-19 related groups in comparison to patients in the unexposed cohort. Across each type of healthcare professional, the SLC subgroup was the most expensive per patient.

**Fig. 2:**
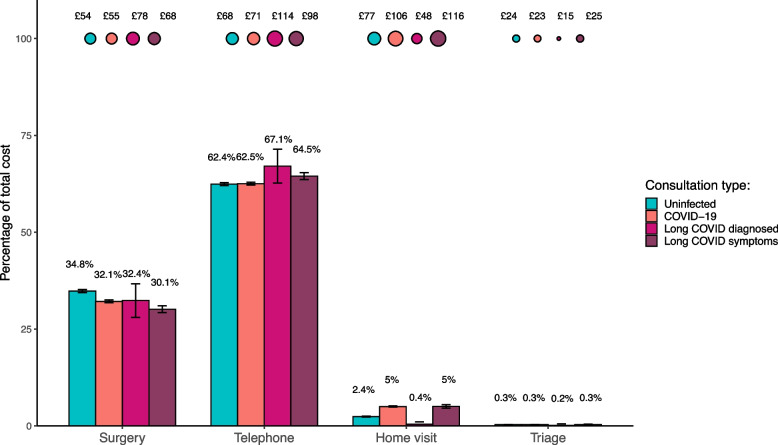
Bubble plot to show the average cost of each consultation type per patient (who had a consultation) between 15^th^ April 2020 and 15^th^ April 2021. Bar chart to show the percentage makeup of each group's total costs by consultation type

For all groups, telephone consultations were the biggest contributor to total costs (over 60%) and were highest in the DLC and SLC subgroups (Fig. 2). By contrast, the burden of in-person consultations on total costs was greatest in the unexposed cohort. Home visits made up a relatively large amount of costs for the exposed cohort and SLC subgroup, in comparison to the other groups. The average incremental costs of home visits for these groups were £19 and £35 higher than those in the unexposed cohort, respectively. Costs stratified by several other demographic factors can be found in Supplementary Fig. 5 and Supplementary Table 13.

The results of the sensitivity analysis are presented in Supplementary Table 14 and Supplementary Fig. 6, which relate to costs among participants who had a minimum of six months of follow-up from their index date.

### National incremental costs

Using estimates of the cumulative incidence of COVID-19 in the ONS COVID-19 Infection Survey and applying an average incremental cost of £2.44 per patient, we estimate the additional primary care consultations cost in the UK associated with long COVID to total £23,382,452 (90% CIs: £21,378,567 to £25,526,052) (Table 3). When applying an average incremental cost of £5.72, based on the sensitivity analysis, we estimate these costs to be £54,814,601 (90% CIs: £50,116,967 to £59,839,762).

**Table 3:** Primary care consultations costs in the UK

	**Population size**	**Cumulative incidence**^**a**^** (%)**	**90% Confidence Interval (LL, UL)**		**Incremental cost (£)**	**Cumulative cases**	**90% Confidence Interval (LL, UL)**		**Proportion**^**b**^** (%)**	**Total incremental cost**	**90% Confidence Interval (LL, UL)**	
Main analysis												
England	56,550,138	70.70	66.00	75.60	2.44	39,980,947.57	37,323,091.08	42,751,904.33	20.86	20,349,662.62	18,996,856.19	21,760,035.27
Scotland	5,466,000	51.50	40.50	63.60	2.44	2,814,990.00	2,213,730.00	3,476,376.00	20.86	1,432,784.87	1,126,753.15	1,769,419.76
Wales	3,169,586	56.00	44.30	69.40	2.44	1,774,968.16	1,404,126.60	2,199,692.68	20.86	903,430.39	714,677.97	1,119,608.38
Northern Ireland	1,895,510	72.20	56.00	90.90	2.44	1,368,558.22	1,061,485.60	1,723,018.59	20.86	696,574.24	540,279.19	876,988.89
UK	67,081,234	68.48	62.61	74.76	2.44	45,939,463.95	42,002,433.28	50,150,991.60	20.86	23,382,452.12	21,378,566.50	25,526,052.31
Sensitivity analysis												
England	56,550,138	70.70	66.00	75.60	5.72	39,980,947.57	37,323,091.08	42,751,904.33	20.86	47,704,946.79	44,533,613.69	51,011,230.23
Scotland	5,466,000	51.50	40.50	63.60	5.72	2,814,990.00	2,213,730.00	3,476,376.00	20.86	3,358,823.55	2,641,404.93	4,147,984.03
Wales	3,169,586	56.00	44.30	69.40	5.72	1,774,968.16	1,404,126.60	2,199,692.68	20.86	2,117,877.81	1,675,392.62	2,624,655.71
Northern Ireland	1,895,510	72.20	56.00	90.90	5.72	1,368,558.22	1,061,485.60	1,723,018.59	20.86	1,632,952.72	1,266,556.13	2,055,892.00
UK	67,081,234	68.48	62.61	74.76	5.72	45,939,463.95	42,002,433.28	50,150,991.60	20.86	54,814,600.86	50,116,967.37	59,839,761.97

^a^ Cumulative incidence of COVID-19 [20]

^b^ The proportion of patients with a history of SARS-CoV-2 infection who had at least one consultation 12 weeks after initial infection. *LL *lower limit of 90% Confidence Intervals, *UL *upper limit of 90% Confidence Intervals

### Risk factor analysis

The results of the log OLS regression model are presented in Table 4. The results showed that having a diagnosis of long COVID or having symptoms of long COVID, were both statistically significant and corresponded to a 43% and 44% increase in primary care consultation costs in comparison to patients with a history of COVID-19 but no record of a long COVID diagnosis or associated symptoms.

**Table 4:** Regression estimates for the log ordinary least squares (OLS) model on primary care consultation costs of patients with COVID-19 at least 12 weeks after infection

Total healthcare cost	(Exp) Coef.^a^	95% Confidence Intervals		*p*-value
Exposure status				
COVID-19 (Reference group)				
Long COVID diagnosis	1.43	1.34	1.52	< 0.001
Symptoms of long COVID	1.44	1.41	1.48	< 0.001
Age (at index date)				
18–29 (Reference group)				
30–39	1.03	1.00	1.07	0.04
40–49	1.05	1.02	1.09	< 0.001
50–59	1.07	1.03	1.11	< 0.001
60–69	1.15	1.10	1.20	< 0.001
70–79	1.37	1.30	1.43	< 0.001
≥ 80	1.49	1.42	1.57	< 0.001
Sex				
Male (Reference group)				
Female	1.04	1.01	1.06	0.01
Ethnic group				
White (Reference group)				
Black	0.94	0.88	0.99	0.03
Other	1.06	0.96	1.18	0.25
Asian	1.00	0.97	1.03	0.97
Mixed	0.99	0.93	1.05	0.64
Ethnicity Missing	0.99	0.96	1.03	0.76
Socioeconomic status (IMD)				
1 (Least deprived) (Reference group)				
2	1.08	1.04	1.13	< 0.001
3	1.07	1.02	1.11	< 0.001
4	1.01	0.96	1.06	0.68
5 (Most deprived)	1.05	1.01	1.10	0.01
IMD Missing	1.00	0.94	1.06	0.92
Smoking status				
Never Smoked (Reference group)				
Ex-Smoker	1.01	0.98	1.04	0.65
Current Smoker	1.00	0.96	1.03	0.83
Smoker Missing	1.00	0.96	1.05	0.88
BMI categories				
Normal weight (Reference group)				
Underweight	1.02	0.95	1.11	0.55
Overweight	0.98	0.95	1.01	0.20
Obese	1.04	1.01	1.07	0.02
BMI Missing	1.09	1.03	1.16	0.01
Charlson Comorbidity Index	1.01	1.01	1.02	< 0.001
GP consultations prior^b^	1.03	1.03	1.04	< 0.001
Nurse consultations prior	1.01	1.01	1.02	< 0.001
Physiotherapist consultations prior	1.04	1.01	1.07	< 0.001
Weeks since index date	1.04	1.04	1.04	< 0.001

^a ^Difference in cost from a one-unit change or in comparison to the reference group – e.g., 1.43 refers to a 43% relative increase in costs due to a one-unit increase or compared to the reference group. These have been adjusted for the covariates in the table in addition to geographic region

^b ^Prior consultations are the sum of the number of consultations a patient had in the 3 to 12 months prior to their index date, with the healthcare professional specified. The coefficients in Table 4 are reported in exponential form. These are interpreted as the percentage change in total cost due to a one unit increase for continuous variables, or the presence of a categorical variable

Older age (49% relative increase in costs in those aged 80 years or older compared to those aged 18 to 29 years), female sex (4% relative increase in costs compared to males), obesity (4% relative increase in costs compared to those of normal weight), comorbidities and frequency of prior consultations were all associated with an increase in the cost of primary care consultations. Those from black ethnic groups had a 6% reduced cost compared to those from white ethnic groups, although no significant differences were seen between white ethnic groups and other minority ethnic groups. While patients from the second, third, and fifth most socioeconomically deprived quintiles had higher costs than those from the least deprived quintile, the differences in these costs did not follow a clear gradient.

## Discussion

Based on over 470,000 non-hospitalised patients with a history of COVID-19 and closely matched individuals with no history of COVID-19, we found that those with a history of infection cost primary care services on average an additional £2.44 per patient for primary care consultations at least 12 weeks after infection. However, this incremental cost could be as high as £5.72 per patient. The incremental costs were significantly higher for those diagnosed with long COVID (£30.52) and those documented as reporting associated symptoms (£57.56). Most of these additional costs were from GP telephone consultations. We estimate that the national costs for primary care consultations to support people with long COVID in the UK are approximately £23 million but may approach £60 million.

Among those with a history of COVID-19, higher consultation costs were associated with having a diagnosis or reporting symptoms of long COVID, older age, being female, and obesity. While the most affluent socioeconomic quintile had lower costs than those from more deprived socioeconomic groups, there was no clear socioeconomic gradient in incremental costs. By contrast, those from black ethnic groups incurred lower costs than those from white ethnic groups, while there was no difference with other ethnic groups.

Using data from the CPRD Aurum database, Whittaker et al. (2021) reported that patients with COVID-19 had significantly higher GP consultation rates, which led to an 18% increase in healthcare utilisation post-infection compared to the 12 months prior [7]. Furthermore, patients with COVID-19 continued to display higher GP consultation rates even four weeks after infection. We further show that this trend continued beyond 12 weeks after SARS-CoV-2 infection and have estimated associated consultation costs.

Koumpias et al. (2022) assessed the healthcare use and costs of over 250,000 patients with a history of COVID-19 using administrative claims data in the United States from March to September 2020 [24]. They found that monthly costs of healthcare resource utilisation increased significantly following COVID-19 compared to prior to infection, with additional costs persisting beyond five months, particularly among adults aged older than 45 years. Their study however did not have a contemporary control group and did not delineate between primary and secondary care services.

Calderón-Moreno et al. (2022) investigated the primary care costs associated with COVID-19 [25]. They assessed 6,286 COVID-19 patients in Aragon, Spanish, estimating an average illness-associated cost of €729.79 per patient. The costing approach was unclear and there are difficulties in comparing healthcare costs between countries, but the study highlighted the significant economic burden of the illness [25]. The authors noted the complications arising from COVID-19, such as respiratory, cardiovascular, and haematological disorders, caused further cost increases, but they did not specifically comment on the costs associated with long COVID.

There is also broader literature on the impact of COVID-19 on the utilisation of primary care resources. For many patients, especially those with less severe illnesses, the pandemic led to a reduction in overall healthcare use, but an increase in the number of non-face-to-face consultations [26]. We similarly found that the increased cost of primary consultations associated with long COVID were driven by an increase in telephone consultations.

We found that adults from black ethnic groups incurred lower costs than those from white ethnic groups, while there was no difference with other ethnic groups. This highlights a potential health inequality, especially given the poorer outcomes (e.g., more hospital admissions, higher mortality rate) following COVID-19 among individuals from black ethnic minority groups [27, 28]. This finding is also highlighted by some existing studies showing the health inequalities in individuals from black and minority ethnic backgrounds within the United Kingdom, which was exposed and exaggerated by the COVID-19 pandemic [29–32]. However, findings on health inequalities and healthcare service use for minority ethnic groups are mixed [31–33], suggesting that more research is needed to explore health and healthcare service use among different ethnic groups in the post-pandemic era in the UK context.

A strength of the study was that the costs associated with long COVID could be isolated by implementing an incremental cost approach using a highly matched comparison group with no prior history of suspected or confirmed COVID-19. The comprehensive matching algorithm, accounting for many relevant variables, successfully balanced demographics, and clinical characteristics between the exposed and unexposed cohorts. This was fundamental to the inferences being made, as except from unobservable factors, the only key difference between the cohorts was the record of SARS-CoV-2 infection [34]. Another strength was the large sample size (i.e., 944,346 patients), which boosted statistical power for our analyses and ensured representative results for the UK population [14, 35].

A key limitation was the lack of long COVID diagnosis in primary care records [18]. Our study incorporated costs for consultations that occurred at least 12 weeks after confirmation of SARS-CoV-2 infection (or matched time point for the unexposed cohort). We inferred that any differences in consultation costs beyond this time point were likely to be attributable to long COVID, given that both cohorts had similar characteristics except for SARS-CoV-2 infection.

The duration of consultations is not well recorded, limiting cost calculations. We used PSSRU’s 2021 Unit costs for primary care consultations with standard durations, but factors like clinician experience and patient characteristics might alter actual durations and consultation costs [36]. Furthermore, when estimating the national costs from consultations associated with long COVID, we assumed that incremental costs would remain constant over the course of the pandemic, which may not necessarily be true as access to primary care changed during this period. Moreover, we extrapolated our findings from data derived primarily from English general practices to the whole of the UK, which assumes that primary care service use and associated costs are the same for different nations of the UK. However, this assumption may not hold if there were significant differences in access to healthcare services and associated costs across the different nations.

We used propensity score matching to reduce confounding, but residual confounding may still affect differences in consultation rates between the exposed and unexposed cohorts. However, we anticipate that residual confounding would be limited in our results, given the wide range of demographic and clinical covariates considered. The subgroup analysis was undertaken without propensity score matching, which increases the likelihood of the comparison groups being different with respect to important confounding factors. This was done to make maximum use of the available data from the defined subgroups. However, we adjusted for a range of relevant confounding factors in the multivariable OLS regression model, which should minimise the impact of confounding in our subgroup analyses of factors influencing incremental consultation costs.

Another limitation is the potential misclassification of individuals in the unexposed cohort due to limited community testing during the pandemic’s first wave [37]. Some members of the unexposed cohort may have had COVID-19 but not been formally tested. We attempted to limit this by excluding patients from the unexposed cohort if they had a record of either suspected or confirmed COVID-19, even in the absence of any confirmatory testing. However, misclassification bias may still be present, leading to an underestimation of the true incremental cost of primary care consultations associated with long COVID.

Our analysis indicates substantial primary care costs to support non-hospitalised patients with long COVID, even when only considering consultation costs. This is at a time of exceptional pressure on health services, including primary care in the UK and worldwide. UK primary care may require £20-£60 million for primary care consultations in patients with long COVID, mostly for remote GP consultations, with similar costs in comparable settings. It should be noted that some non-hospitalised patients with COVID-19 might require secondary care referral, causing further costs not considered here. Overall, significant investment globally is needed for primary care services to address the complex care needs and ongoing symptoms of non-hospitalised patients. Training allied healthcare professionals to support this care and implementing guidelines for long COVID diagnosis and care [38], could potentially reduce these costs.

Our analysis also indicates significant additional primary care costs for patients with a history of COVID-19 and reporting relevant symptoms, without a formal long COVID diagnosis. Furthermore, certain population subgroups amongst those with a history of COVID-19 can incur increased costs, such as the elderly, females, and those with obesity. Additionally, those from black ethnic groups may be underusing primary care services for long COVID symptoms, representing a potential health inequity. These factors should be considered by health service commissioners, managers and providers when designing and resourcing long COVID services in primary care as well as planning for similar future pandemic viruses.

Our study provides a foundation in methods and cost estimates for future cost analyses and economic evaluations on long COVID, with lessons for future pandemic planning, including the need for careful planning for the longer-term impacts of pandemics. Future research should focus on updating this analysis to capture longer-term patient data and costs, evaluate the impact of long COVID on prescription drug costs, assess secondary care costs, assess out-of-pocket costs, and explore methods to better capture costs specifically attributable to long COVID.

## Conclusion

The support of non-hospitalised individuals with long COVID in primary care is likely to be substantial, requiring significant healthcare investment and planning. This particularly applies to patients who have been formally diagnosed with long COVID, those without a long COVID diagnosis but with a history of COVID-19 and reporting related symptoms, the elderly, females, and those with obesity. Inequalities in access to primary care services for long COVID support require further exploration and need to be addressed.

### Supplementary Information

**Additional file 1.** Supplementary material.

## Data Availability

Following protocol approval from the MHRA Independent Scientific Advisory Committee, access to anonymised patient data from the Clinical Practice Research Datalink (CPRD) is authorised based on the data sharing agreement with specific terms and conditions of usage. Therefore, the dataset used in this study is not available to the public. Requests for access to the data used for this study will need to be directed to CPRD.
