# Supplementary material for: The cost of primary care consultations associated with long COVID in non-hospitalised adults: a retrospective cohort study using UK primary care data
Source: BMC Prim Care. 2023 Nov 20;24:245. doi: 10.1186/s12875-023-02196-1 (PMC10662438; doi:10.1186/s12875-023-02196-1)
Supplement: Supplementary file 1 — Additional file 1. Supplementary material. [file 12875_2023_2196_MOESM1_ESM.docx]

**Supplementary material**

**The economic cost of primary care consultations associated with long Covid in non-hospitalised adults: a retrospective cohort study using UK primary care data**

Authors: Jake Tufts, Naijie Guan, Dawit T Zemedikun, Anuradhaa Subramanian, Krishna Gokhale, Puja Myles, Tim Williams, Tom Marshall, Melanie Calvert, Karen Matthews, Krishnarajah Nirantharakumar, Louise Jackson, Shamil Haroon

### **Table of Contents**

[**Table of Contents** 2](#_Toc138231873)

[Section 1. Supplementary Methods and Results 3](#_Toc138231874)

[**Appendix S1.** Propensity score matching 3](#_Toc138231875)

[**Appendix S2.** Costing method 4](#_Toc138231876)

[**Appendix S3.** Sensitivity analysis 6](#_Toc138231877)

[Section 2. Supplementary Figures 8](#_Toc138231878)

[**Supplementary Figure 1.** Timeline of the dates and time periods of interest throughout the study 8](#_Toc138231879)

[**Supplementary Figure 2.** Flow chart of the selection of the study population 9](#_Toc138231880)

[**Supplementary Figure 3**. Kernel density plot before and after propensity score matching to show the comparison in the distribution of the propensity scores between the exposed and unexposed groups 10](#_Toc138231881)

[**Supplementary Figure 4.** Standardised differences across covariates in the matched groups 11](#_Toc138231882)

[**Supplementary Figure 5.** Heatmap of the difference in mean cost per patient (who had a consultation) between the unexposed patients and COVID-19 groups’ patients, stratified by demographic characteristics 12](#_Toc138231883)

[**Supplementary Figure 6.** Heatmap of the difference in mean cost per patient (who had a consultation) between the unexposed patients and COVID-19 groups’ patients, stratified by demographics. – Sensitivity analysis 13](#_Toc138231884)

[Section 3. Supplementary Tables 14](#_Toc138231885)

[**Supplementary Table 1.** Table of all descriptions of COVID-19 and relevant SNOMED CT codes used to define whether a patient had COVID-19 14](#_Toc138231886)

[**Supplementary Table 2.** Table of explanatory variables included in the study 15](#_Toc138231887)

[**Supplementary Table 3.** Study group definitions and titles 17](#_Toc138231888)

[**Supplementary Table 4.** Table of Long COVID symptoms classified by WHO Symptoms must be shown by a COVID-19 patient 12 weeks after their index date 18](#_Toc138231889)

[**Supplementary Table 5.** Table of the CPRD Aurum codes and descriptions of healthcare professionals included in the study, grouped by the healthcare professional category 19](#_Toc138231890)

[**Supplementary Table 6.** Table of the CPRD Aurum codes and descriptions of consultation types included in the study, grouped by the consultation type category 20](#_Toc138231891)

[**Supplementary Table 7.** Table of costs per length of time for each healthcare professional 24](#_Toc138231892)

[**Supplementary Table 8.** Table of the length of consultation for each healthcare professional by consultation type 25](#_Toc138231893)

[**Supplementary Table 9.** Example of how the cost of a surgery consultation by a Nurse from a GP practice is calculated 26](#_Toc138231894)

[**Supplementary Table 10.** Comorbidities at baseline in matched exposed and unexposed groups 27](#_Toc138231895)

[**Supplementary Table 11.** Incremental cost OLS regression estimates for primary care healthcare cost associated with Long COVID 30](#_Toc138231896)

[**Supplementary Table 12.** Baseline characteristics for the COVID patients used in the part 2 regression analysis (incurred a positive cost) 31](#_Toc138231897)

[**Supplementary Table 13.** The mean cost of healthcare professional consultations for COVID-19 patients, stratified by subgroups of patients 35](#_Toc138231898)

[**Supplementary Table 14.** Estimates of first three months (after index date + 12 weeks) of primary care resource use and costs associated with Long COVID, for patients who had at least three months of follow-up. (Sensitivity analysis) 37](#_Toc138231899)

## **Section 1. Supplementary Methods and Results**

### **Appendix S1.** Propensity score matching

Propensity score matching (1:1) was used to closely match patients from the exposed and unexposed cohorts on several important confounding factors including age, sex, body mass index (BMI), smoking status, ethnic group, socioeconomic status (Index of Multiple Deprivation, IMD),^1^ index date, follow-up time from the index date, registered general practice, the number of primary care consultations in the 12 months prior to the index date (to account for informed presence bias),^2^ comorbidities and geographical region (Supplementary Table 2).

Propensity scores were derived from a logistic regression model of the probability of having a COVID-19 diagnosis as a function of 10 categories of covariates (Supplementary Table 2), with a calliper width of one-quarter of the standard deviation of the propensity score (0.04).^3^ If matching is implemented properly, it can be assumed that the only systematic difference between the exposed and unexposed cohorts is the diagnosis of COVID-19, identifying the costs specifically caused by the disease.^4^ The matching performance was evaluated by comparing kernel density plots before and after matching to check the distribution of propensity scores and using the standardised differences between the groups for each variable, where a value greater than 0.1 was considered to indicate imbalance in baseline characteristics.

### **Appendix S2.** Costing method

The costs of these consultations were estimated. Costs were assigned to the most specific cost data available. Unit costs for healthcare resources were taken from the Personal Social Services Research Unit’s (PSSRU) Unit Costs of Health and Social Care 2021, to account for inflation and variations in pricing over time, and to represent the cost perspective of the UK National Health Service (NHS).^5^ The hourly cost was available for each healthcare professional and the average consultation duration was used to calculate consultation costs. Multiple consultations on the same day with the same healthcare professional were counted as a single consultation. Further details are provided in Supplementary Tables 7-9.

The incremental cost of primary care consultations between the exposed and unexposed cohorts was then estimated. The primary costing method was an ‘incremental cost’ approach. Bottom-up costing was adopted to estimate the healthcare use, with each patient’s resource utilisation calculated using individual-level data on consultations from CPRD Aurum.^6^ The healthcare utilisation was then multiplied by its respective unit cost and summed to obtain a patient’s total cost as follows:

$$Total patient cost=\sum_{i} \left( Resource utilisation \right)_{j} \times\left( Resource \cos t \right)_{j}$$

$i=Patient, j=Type of resource$ (1)

The incremental cost associated with long COVID was then obtained by subtracting the sum of each patient’s total healthcare cost between the matched groups, 12 weeks after the index date:

$$Incremental cost{}_{Long COVID}=\sum_{i}^{N} \left( Total cost \right)_{i, exposed} -\sum_{i}^{N} \left( Total cost \right)_{i, unexposed}$$

$i=Patient, N=Total number of patients$ (2)

Usually, a cost of illness (COI) study includes a full year of data on all patients, as otherwise different follow-up times could be a major confounder. However, time since infection was included as a variable within the propensity score matching model to ensure similar follow-up time distributions in both cohorts.

To estimate the national incremental costs attributed to primary care consultations for non-hospitalised patients with long COVID across the whole UK population, cumulative COVID-19 incidence estimates produced by the UK Office for National Statistics (ONS) in the COVID-19 Infection Survey were used. This was done by multiplying the population size by the cumulative incidence of COVID-19, as well as the 90% credible intervals. This provided the cumulative frequency of COVID-19. This was multiplied by the proportion of non-hospitalised patients with COVID-19 who had received at least one primary care consultation within the follow-up period in our study data. This value was then multiplied by the incremental cost estimated in the first part of the analysis to provide the national incremental cost of primary care consultations associated with long COVID. This was done for England, Wales, Scotland, and Northern Ireland, and for the total UK population. This was done separately using the estimated incremental costs from both the primary and sensitivity analyses. We assumed that the incremental costs would remain constant throughout the pandemic.

### **Appendix S3.** Sensitivity analysis

A sensitivity analysis was conducted to assess the assumption that follow-up time does not confound the costs. The dataset was restricted to only patients who had at least six months of follow-up time from their index date. Only cost data from three to six months from the index date was included in the sensitivity analysis.

The results of the sensitivity analysis are presented in Supplementary Table 14 and Supplementary Figure 6, which relate to costs among participants who had a minimum of six months of follow-up from their index date. These followed a similar trend to the cost per person year estimates in the main analysis although the overall incremental costs were higher. The exposed cohort was more costly than the unexposed cohort, with an incremental cost of £5.72 per patient. Patients in the DLC subgroup were the most expensive, followed by the SLC subgroup, with incremental costs per patient of £68.55 and £46.98, respectively when compared to the unexposed cohort.

**References:**

1. Ministry of Housing, Communities, Local Government. English indices of deprivation 2019. GOV.UK; 2019. [cited 2022 Nov 20]. Available from: <https://www.gov.uk/government/statistics/english-indices-of-deprivation-2019>
2. Goldstein BA, Bhavsar NA, Phelan M, Pencina MJ. Controlling for informed presence bias due to the number of health encounters in an electronic health record. American journal of epidemiology. 2016;184(11):847–55. DOI: <http://dx.doi.org/10.1093/aje/kww112>
3. Stuart EA, Rubin DB. Best practices in quasi-experimental designs. Best practices in quantitative methods. 2008:155-76. DOI: <https://dx.doi.org/10.4135/9781412995627.d14>
4. Akobundu E, Ju J, Blatt L, Mullins CD. Cost-of-illness studies. Pharmacoeconomics. 2006 Sep;24(9):869-90. DOI:<https://do>i.org/10.2165/00019053-200624090-00005
5. Personal Social Services Unit. Unit Costs of Health and Social Care 2021 [Internet]. 2022 Jan 31. [Cited 22/04/2022]. Available from: DOI: [10.22024/UniKent/01.02.92342](https://doi.org/10.22024/UniKent%2F01.02.92342)
6. Pagano E, Brunetti M, Tediosi F, Garattini L. Costs of diabetes. Pharmacoeconomics. 1999 Jun;15(6):583-95. DOI: <https://doi.org/10.2165/00019053-199915060-00006>

## **Section 2. Supplementary Figures**

### **Supplementary Figure 1.** Timeline of the dates and time periods of interest throughout the study

472,173 eligible exposed patients

8,549,416 unexposed patients

Excluded unexposed patients because:

- Hospitalised within the exclusion timer period.
  - 33,043
- Transferred out of practice before index date.
  - 4,996
- Died before index date.
  - 266
- Transferred out of practice for reasons other than death.
  - 399,152

8,118,797 eligible unexposed patients

472,173 matched unexposed patients

472,173 eligible exposed patients

3,871 exposed patients with a record of diagnosed Long COVID

30,174 exposed patients with reported WHO symptoms of Long COVID after 12 weeks since index date, but without a record of Long COVID diagnosis

472,173 matched exposed patients

Excluded 7,646,624 unexposed patients due to 1:1 propensity score matching

66,303 exposed patients

444 Long COVID diagnosed

10,310 symptoms of Long COVID

64,604 unexposed patients

Excluded 405,870 exposed patients, 407,569 unexposed patients, 3,427 Long COVID diagnosed patients and 19,864 symptoms of Long COVID patients due to them not having 3 months of follow-up after 12 weeks post index date.

**Main analysis**

**Sensitivity analysis**

### **Supplementary Figure 2.** Flow chart of the selection of the study population

The kernel density plots show that the matching was well-balanced because the density estimation lines for the two groups coincide. The standardised differences across covariates were less than 0.1 for all variables after matching (Supplementary Figure 5), which is further evidence of balanced matching.

### **Supplementary Figure 3**. Kernel density plot before and after propensity score matching to show the comparison in the distribution of the propensity scores between the exposed and unexposed groups

### **Supplementary Figure 4.** Standardised differences across covariates in the matched groups

**

### **Supplementary Figure 5.** Heatmap of the difference in mean cost per patient (who had a consultation) between the unexposed patients and COVID-19 groups’ patients, stratified by demographic characteristics

*Note: Yellow and red represent a greater cost difference from the unexposed.*

### **Supplementary Figure 6.** Heatmap of the difference in mean cost per patient (who had a consultation) between the unexposed patients and COVID-19 groups’ patients, stratified by demographics. – Sensitivity analysis

## **Section 3. Supplementary Tables**

### **Supplementary Table 1.** Table of all descriptions of COVID-19 and relevant SNOMED CT codes used to define whether a patient had COVID-19

| **Description** | **SNOMED CT Code** |
| --- | --- |
| Detection of 2019 novel coronavirus using polymerase chain reaction technique | 1240511000000100 |
| Detection of 2019-nCoV (novel coronavirus) using polymerase chain reaction technique | 1240511000000100 |
| Detection of SARS-CoV-2 (severe acute respiratory syndrome coronavirus 2) using polymerase chain reaction technique | 1240511000000100 |
| Detection of Wuhan 2019-nCoV (novel coronavirus) using polymerase chain reaction technique | 1240511000000100 |
| Wuhan 2019-nCoV (novel coronavirus) detected | 1240581000000100 |
| 2019-nCoV (novel coronavirus) detected | 1240581000000100 |
| 2019 novel coronavirus detected | 1240581000000100 |
| SARS-CoV-2 (severe acute respiratory syndrome coronavirus 2) detected | 1240581000000100 |
| SARS-CoV-2 (severe acute respiratory syndrome coronavirus 2) detection result positive | 1240581000000100 |
| SARS-CoV-2 (severe acute respiratory syndrome coronavirus 2) RNA (ribonucleic acid) detection result positive | 1240581000000100 |
| COVID-19 detected | 1240581000000100 |
| COVID-19 confirmed by laboratory test | 1300721000000100 |
| SARS-CoV-2 (severe acute respiratory syndrome coronavirus 2) RNA (ribonucleic acid) qualitative existence in specimen | 1321301000000100 |
| SARS-CoV-2 (severe acute respiratory syndrome coronavirus 2) antigen detection result positive | 1322781000000100 |
| 2019-nCoV (novel coronavirus) antigen detection result positive | 1322781000000100 |
| SARS-CoV-2 (severe acute respiratory syndrome coronavirus 2) RNA (ribonucleic acid) detection result positive | 1324601000000100 |
| 2019-nCoV (novel coronavirus) ribonucleic acid detected | 1324601000000100 |
| SARS-CoV-2 (severe acute respiratory syndrome coronavirus 2) RNA (ribonucleic acid) detection result positive at the limit of detection | 1324881000000100 |
| 2019-nCoV (novel coronavirus) detection result positive at the limit of detection | 1324881000000100 |
| Detection of SARS-CoV-2 (severe acute respiratory syndrome coronavirus 2) antigen | 871553007 |
| Detection of ribonucleic acid of 2019 novel coronavirus in nasopharyngeal swab | 871556004 |
| Detection of RNA (ribonucleic acid) of SARS-CoV-2 (severe acute respiratory syndrome coronavirus 2) in nasopharyngeal swab | 871556004 |
| Detection of RNA (ribonucleic acid) of SARS-CoV-2 (severe acute respiratory syndrome coronavirus 2) in oropharyngeal swab | 871557008 |
| Detection of RNA (ribonucleic acid) of SARS-CoV-2 (severe acute respiratory syndrome coronavirus 2) using polymerase chain reaction | 871560001 |
| Detection of ribonucleic acid of COVID-19 using polymerase chain reaction | 871560001 |

### **Supplementary Table 2.** Table of explanatory variables included in the study

| **Variable** | **Description** | **Specification** | **Models** |
| --- | --- | --- | --- |
| Age | Age in years at index date | Continuous | * f |
| Age group | Age categorised into 8 groups:  18-29, 30-49, 50-59, 60-69, 70-79, 80+ | Derived categorical | g |
| Sex | Male or Female | Binary | * g f |
| BMI category | Underweight <18.5 kg/m2,  Normal weight 18.5-24 kg/m2  Overweight 25-29 kg/m  Obese ≥30 kg/m2  BMI Missing | Derived categorical | * g f |
| Index of Multiple Deprivation | Measure of socio-economic status from 1 to 5:  1 – Least deprived  5 – Most deprived  IMD missing | Categorial | * g f |
| Smoking status | Never smoked  Ex-smoker  Current smoker  Smoker Missing | Categorial | * g f |
| Ethnicity | White  Black  Mixed  Asian  Other  Ethnicity Missing | Categorical | * g f |
| Time since index | Number of weeks from index date to patient’s end of study period | Derived continuous | * f |
| Practice ID | Indicates which practice a patient belongs to. | Categorial | * |
| Prior healthcare utilisation | Number of consultations had with a healthcare professional in the 3 to 12 months prior to a patient’s index date:  GP  Nurse  Physiotherapist | Continuous | * f |

**Supplementary Table 2.** Table of explanatory variables included in the study (Continued)

| **Variable** | **Description** | **Specification** | **Models** |
| --- | --- | --- | --- |
| 87 Comorbidities | Chronic health conditions, one category with patient numbers ≤5 was suppressed in accordance with CPRD guidelines for data protection. The list of comorbidities can be found in Supplementary Table 11. | Binary | * |
| Charlson Comorbidity Index  (CCI) | Using a CCI R package, CCI was calculated for each patient. 17 comorbidities are included and are given different scores dependant on the range a patient has. | Derived continuous | f |
| Region | 11 regions from England and Northern Ireland:  Northeast, North West, East Midlands, West Midlands, South East, South West, East of England, South Central, London, Northern Ireland | Categorial | g f |

*Note: * Included in propensity score matching. g: Included in stratification of costs. f: Included in the regression specification.*

### **Supplementary Table 3.** Study group definitions and titles

|  | **Main groups** | | **Sub-groups**  (Patients with a diagnosis of COVID-19…) | |
| --- | --- | --- | --- | --- |
| **Group title** | **Unexposed group** | **Exposed group** | **Diagnosed Long COVID (DLC) group** | **Symptoms of Long COVID (SLC) group** |
| **Definition** | Patients without a diagnosis of COVID-19 | Patients with a diagnosis of COVID-19 | … and a diagnosis of Long COVID | … and show at least one of the WHO symptoms of Long COVID after 12 weeks post infection |

### **Supplementary Table 4.** Table of Long COVID symptoms classified by WHO Symptoms must be shown by a COVID-19 patient 12 weeks after their index date

| **Symptom** | **SNOMED CT Code** |
| --- | --- |
| Anosmia | 44169009 |
| Fatigue | 84229001 |
| Fever | 386661006 |
| Palpitations | 80313002 |
| Blurred vision/diplopia | 24982008 |
| Chest pain | 29857009 |
| Shortness of breath | 267036007 |
| Paraesthesia | 91019004 |
| Diarrhoea | 62315008 |
| Headache | 25064002 |
| Allergies | 370860007 |
| Insomnia | 193462001 |
| Constipation | 14760008 |
| Joint pain | 57676002 |
| Difficulty thinking | 285257002 |
| Abdominal pain | 21522001 |
| Gastritis | 4556007 |
| Dizziness | 404640003 |
| Menorrhagia | 386692008 |
| Hyperacusis | 25289003 |
| Muscle pain | 68962001 |
| Amnesia | 48167000 |
| Muscle cramping | 55300003 |
| Gastric reflux | 225587003 |
| Depression | 255339005 |
| Dysgeusia | 271801002 |
| Anxiety | 48694002 |
| Menstrual changes | 80182007 |
| Pre-menstrual syndrome | 289896006 |
| Excessive sleep | 77692006 |
| Hearing loss | 15188001 |
| Post exert fatigue | 444042007 |

### **Supplementary Table 5.** Table of the CPRD Aurum codes and descriptions of healthcare professionals included in the study, grouped by the healthcare professional category

| **CPRD Aurum codes: jobcatid** | **Description** |
| --- | --- |
| **GP** |  |
| 4 | General Medical Practitioner |
| 5 | Salaried General Practitioner |
| 15 | Associate Practitioner - General Practitioner |
| 24 | GP Registrar |
| 31 | Sessional GP |
| 181 | Locum GP |
| 183 | Assistant GP |
| **General Nurse** |  |
| 9 | Community Nurse |
| 48 | Enrolled Nurse |
| 52 | Community Mental Health Nurse |
| 61 | Sister/Charge Nurse |
| **Staff Nurse** |  |
| 47 | Staff Nurse |
| 59 | Student Practice Nurse |
| 115 | Student District Nurse |
| 169 | Student Community Mental Health Nurse |
| 191 | Student Occupational Health Nurse |
| **Specialist Nurse** |  |
| 27 | Specialist Nurse Practitioner |
| 55 | Associate Practitioner - Nurse |
| **Nurse consultant** |  |
| 33 | Nurse Consultant |
| **Nurse Manager** |  |
| 60 | Nurse Manager |
| **Physiotherapist Consultant** |  |
| 106 | Physiotherapist Consultant |
| **Physiotherapist Specialist Practitioner** |  |
| 80 | Physiotherapist Specialist Practitioner |
| **Physiotherapist Manager** |  |
| 136 | Physiotherapist Manager |
| **Physiotherapist** |  |
| 34 | Physiotherapist |
| 77 | Student Physiotherapist |

### **Supplementary Table 6.** Table of the CPRD Aurum codes and descriptions of consultation types included in the study, grouped by the consultation type category

| **CPRD Aurum codes: Consultation Source Id** | **Description** |
| --- | --- |
| **Surgery** |  |
| 751 | G.P. Evening Surgery |
| 4221 | Surgery consultation |
| 3509 | Nurse Surgery |
| 10310 | Surgery Clinic |
| 5266 | GP Surgery |
| 2706 | GP Surgery |
| 4231 | GP Surgery |
| 754 | GP Surgery |
| 7632 | GP Surgery |
| 7659 | GP Surgery |
| 7732 | GP Surgery |
| 9361 | Surgery or Clinic |
| 2711 | G.P Surgery (Pm) |
| 4215 | Surgery Attendance |
| 1865 | Branch Surgery |
| 2719 | G.P. Morning Surgery |
| 3505 | Nurse Practitioner Surgery |
| 6963 | Walk-In Surgery |
| 426 | Contact method: G.P.Surgery |
| 2580 | Emergency Gp Surgery |
| 5252 | G.P.Surgery Urgent Consultation |
| 10303 | Surgery |
| 9551 | Unbooked Clinic |
| 3499 | Nurse Assessment Clinic |
| 6140 | P.Nurse Clinic |
| 364 | Clinic NHS |
| 366 | Clinic note |
| 3346 | Minor Operations Clinic |
| 3503 | Nurse Minor Illness Clinic |
| 5093 | Emergency Nurse Clinic |
| 6236 | Practice Nurse Clinic |
| 7240 | Clinic Premises |
| 397 | Community Clinic |
| 10310 | Surgery Clinic |
| 4773 | Clinic |
| 5952 | Nurse's Treatment Room Clinic |
| 3347 | Minor Ops Clinic |

**Supplementary Table 6.** Table of the CPRD Aurum codes and descriptions of consultation types included in the study, grouped by the consultation type category **(Continued)**

| **CPRD Aurum codes: Consultation Source Id** | **Description** |
| --- | --- |
| 3501 | Nurse Flu Clinic |
| 3516 | Nurses' Flu Clinic |
| 7395 | Diabetic Clinic |
| 8812 | Out Of Hours Visit |
| 3631 | Out Of Hours Night Visit |
| 3627 | Out Of Hours Gp Visit |
| 5869 | Night Visit |
| 6087 | Out of hours, Practice |
| 16305 | Out of Hours |
| 6034 | Out of Hours |
| 6052 | Out of Hours |
| 6056 | Out of Hours |
| 6062 | Out of Hours |
| 6063 | Out of Hours |
| 8794 | Out of Hours |
| 8795 | Out of Hours |
| 8796 | Out of Hours |
| 3617 | Out of Hours |
| 3618 | Out of Hours |
| 8503 | Out of Hours |
| 8504 | Out of Hours |
| 8505 | Out of Hours |
| 8506 | Out of Hours |
| 6064 | Out of Hours |
| 6065 | Out of Hours |
| 6066 | Out of Hours |
| 3614 | Out of Hours |
| 3615 | Out of Hours |
| 3616 | Out of Hours |
| 8799 | Out of hours consultation at surgery |
| 3632 | Out Of Hours Other Practice |
| 9907 | Practice Nurse |
| 2810 | GP Practice |
| 5875 | Night visit, practice |
| 6236 | Practice Nurse Clinic |
| 9203 | Seen by Practice Nurse |
| 745 | G P Consultation |
| 421 | Consultation |

**Supplementary Table 6.** Table of the CPRD Aurum codes and descriptions of consultation types included in the study, grouped by the consultation type category **(Continued)**

| **CPRD Aurum codes: Consultation Source Id** | **Description** |
| --- | --- |
| 3626 | Out Of Hours GP Service |
| 9633 | Walk In Centre |
| 4523 | Walk-in clinic |
| 6963 | Walk-In Surgery |
| 5149 | Face to face consultation |
| 10474 | Third party consultation |
| **Home visit** |  |
| 7883 | Home of Patient |
| 3628 | Out Of Hours Home Visit |
| 7885 | Home Visit |
| 984 | Home Visit - In Surgery Hours |
| 5464 | Home visit note |
| 10627 | Visit-Home |
| 8348 | Normal Home Visit (08:00 - 11:00) |
| 8400 | Nursing Home |
| 9060 | Residential Home |
| 3430 | Night Visit - patient's home |
| 3431 | Night Visit - patient's home |
| 3432 | Night Visit - patient's home |
| 7358 | Daytime Visits patients home |
| 6517 | Seen in own home |
| 3438 | Night Visit patients home |
| 9061 | Residential home visit note |
| 8703 | Nursing home visit note |
| 8527 | Out of hours, Non-Practice |
| 3514 | Nurse Visit |
| 3440 | Night visit, Local rota |
| **Telephone** |  |
| 3506 | Nurse Practitioner Telephone Advice |
| 3640 | Out Of Hours-Telephone Advice |
| 4293 | Telephone Consultation |
| 6731 | Telephone call to relative/carer |
| 3639 | Out Of Hours Telephone Contact/Advice |
| 4289 | Telephone Appt |
| 10359 | Telephone |
| 2527 | Duty Doctor Telephone |
| 3636 | Out Of Hours Telephone Advice |
| 3638 | Out Of Hours Telephone Consultation |

**Supplementary Table 6.** Table of the CPRD Aurum codes and descriptions of consultation types included in the study, grouped by the consultation type category **(Continued)**

| **CPRD Aurum codes: Consultation Source Id** | **Description** |
| --- | --- |
| 5040 | Duty Telephone Appt |
| 6728 | Telephone call from a patient |
| 6730 | Telephone call to a patient |
| 3637 | Out Of Hours Telephone Call |
| 4298 | Telephone encounter |
| 6727 | Telephone Call |
| 9417 | Telephone call from relative/carer |
| 4283 | Telephone Advice |
| 4302 | Telephone Surgery |
| **Triage** |  |
| 9433 | Telephone Triage by Doctor |
| 4303 | Telephone Triage |
| 3510 | Nurse Surgery Triage |
| 4271 | Telephone (Triage) |
| 4394 | Triage By Phone |
| 10510 | Triage |
| 3511 | Nurse Triage |
| 3512 | Nurse Triage Clinic |
| 3513 | Nurse Triage Consultation |
| 3512 | Nurse Triage Clinic |
| 9433 | Telephone Triage by Doctor |
| 4303 | Telephone Triage |
| 6751 | Telephone triage encounter |

### **Supplementary Table 7.** Table of costs per length of time for each healthcare professional

| **Healthcare professional** | **2021 cost per length of consultation** | **Source (PSSRU UCHSC*)** |
| --- | --- | --- |
| Physiotherapist | £65 per hour (Band 7) | Chapter 9 (2021) |
| Physiotherapist Specialist Practitioner | £65 per hour (Band 7) |  |
| Physiotherapist Manager | £65 per hour (Band 7) |  |
| Physiotherapist Consultant | £88 per hour (Band 8) |  |
| GP | £34 per 9.22 min | Chapter 10.3b (2021) |
| Nurse GP Practice | £42 per hour | Chapter 10.2 (2021) |
| Staff Nurse | £44 per hour (Band 5) | Chapter 10.1 (2021) |
| Specialist Nurse Practitioner | £66 per hour (Band 7) |  |
| Nurse Manager | £66 per hour (Band 7) |  |
| Nurse Consultant | £146 per hour (Band 9) |  |

*Note: * PSSRU - Personal Social Services Research*

*UCHSC - Unit’s Unit Costs of Health and Social Care*

### **Supplementary Table 8.** Table of the length of consultation for each healthcare professional by consultation type

| **Healthcare professional** | **Consultation type** | **Length of consultation** | **Source (PSSRU UCHSC)** |
| --- | --- | --- | --- |
| Physiotherapists | Surgery | 1 hour | Chapter 9 (2021) |
| GP | Surgery | 9.22 min | Chapter 10.3b (2021)  Chapter 10.8a (2012)  Chapter 10.5 (2021) |
|  | Home visit | 11.4 min |  |
|  | Telephone | 7.1 min |  |
|  | Triage | 4 min |  |
| Nurse | Surgery | 15.5 min | Chapter 10.2 (2021)  Chapter 10.7 (2012)  Chapter 10.5 (2021) |
|  | Home visit | 25 min |  |
|  | Telephone | 6.56 min |  |
|  | Triage | 6.56 min |  |

### **Supplementary Table 9.** Example of how the cost of a surgery consultation by a Nurse from a GP practice is calculated

**Nurse GP Practice cost per hour = £43**

**Nurse giving a surgery consultation = 15.5 mins**

**Cost for one Nurse from a GP practice giving a surgery consultation**

**= £43/60mins * 15.5 mins = £11.10**

### **Supplementary Table 10.** Comorbidities at baseline in matched exposed and unexposed groups

| **Comorbidity** | **Unexposed** | **Exposed** |
| --- | --- | --- |
| Cancer | 20,571 (4.4) | 20,621 (4.4) |
| Arrhythmia | 33,306 (7.1) | 33,575 (7.1) |
| Atrial Fibrillation | 9,185 (1.9) | 9,171 (1.9) |
| Hypertension | 72,412 (15.3) | 71,916 (15.2) |
| Heart Failure | 4,273 (0.9) | 4,176 (0.9) |
| Ischaemic Heart Disease | 13,219 (2.8) | 13,222 (2.8) |
| Myocardial Infarction | 6,599 (1.4) | 6,573 (1.4) |
| Valvular Heart Disease | 5,976 (1.3) | 5,923 (1.3) |
| Cardiomyopathy | 1,236 (0.3) | 1,213 (0.3) |
| Congenital Heart Disease | 2,905 (0.6) | 2,876 (0.6) |
| Peripheral Vascular Disease | 4,346 (0.9) | 4,470 (0.9) |
| Aortic Aneurysm | 960 (0.2) | 919 (0.2) |
| Transient Ischaemic Attack | 4,083 (0.9) | 4,192 (0.9) |
| Ischaemic Stroke | 2,203 (0.5) | 2,297 (0.5) |
| Haemorrhagic Stroke | 1,263 (0.3) | 1,331 (0.3) |
| Stroke Unspecified | 3,549 (0.8) | 3,640 (0.8) |
| Eczema | 92,173 (19.5) | 91,554 (19.4) |
| Psoriasis | 19,491 (4.1) | 19,392 (4.1) |
| Autoimmune Skin Conditions | 6,359 (1.3) | 6,421 (1.4) |
| Acne | 65,310 (13.8) | 64,289 (13.6) |
| Hay Fever | 86,186 (18.3) | 85,419 (18.1) |
| Chronic Sinusitis | 8,306 (1.8) | 8,237 (1.7) |
| Deafness | 5,120 (1.1) | 5,193 (1.1) |
| Blindness | 2,086 (0.4) | 2,159 (0.5) |
| Cataract | 15,743 (3.3) | 16,043 (3.4) |
| Glaucoma | 4,865 (1.0) | 4,955 (1.0) |
| Age-related Macular Degeneration | 3,160 (0.7) | 3,322 (0.7) |
| Diabetic Retinopathy | 12,748 (2.7) | 12,662 (2.7) |
| Inflammatory Eye Disease | 8,073 (1.7) | 8,118 (1.7) |
| Peptic Ulcer | 6,385 (1.4) | 6,426 (1.4) |
| Inflammatory Bowel Disease | 4,767 (1.0) | 4,633 (1.0) |
| Irritable Bowel Syndrome | 33,647 (7.1) | 33,428 (7.1) |
| Hepatitis B | 1,405 (0.3) | 1,362 (0.3) |
| Hepatitis C | 829 (0.2) | 815 (0.2) |
| Alcohol Related Chronic Liver Disease | 852 (0.2) | 860 (0.2) |
| Chronic Liver Disease (All) | 13,489 (2.9) | 13,332 (2.8) |
| Non-Alcoholic Fatty Liver Disease | 6,171 (1.3) | 6,066 (1.3) |
| Diverticular Disease | 13,687 (2.9) | 13,806 (2.9) |

**Supplementary Table 10.** Comorbidities at baseline in matched exposed and unexposed groups **(Continued)**

| **Comorbidity** | **Unexposed** | **Exposed** |
| --- | --- | --- |
| Coeliac Disease | 2,094 (0.4) | 2,066 (0.4) |
| Chronic Pancreatitis | 372 (0.1) | 364 (0.1) |
| Endometriosis | 6,873 (1.5) | 6,916 (1.5) |
| Poly Cystic Ovarian Syndrome | 11,673 (2.5) | 11,667 (2.5) |
| Low Haemoglobin | 25,478 (5.4) | 25,407 (5.4) |
| Venous Thromboembolism | 9,289 (2.0) | 9,181 (1.9) |
| Coagulopathy | 5,463 (1.2) | 5,360 (1.1) |
| Pernicious Anaemia | 1,242 (0.3) | 1,236 (0.3) |
| Depression | 105,548 (22.4) | 104,666 (22.2) |
| Anxiety | 96,512 (20.4) | 96,129 (20.4) |
| Serious Mental Illness | 5,152 (1.1) | 5,167 (1.1) |
| Substance Misuse | 8,275 (1.8) | 8,227 (1.7) |
| Alcohol Misuse | 24,173 (5.1) | 24,162 (5.1) |
| Attention Deficit Hyperactivity Disorder | 2,462 (0.5) | 2,433 (0.5) |
| Eating Disorder | 4,251 (0.9) | 4,187 (0.9) |
| Learning Disability | 4,318 (0.9) | 4,329 (0.9) |
| Alzheimer’s | 3,093 (0.7) | 3,655 (0.8) |
| Vascular Dementia | 1,521 (0.3) | 1,903 (0.4) |
| Dementia Unspecified | 6,768 (1.4) | 7,886 (1.7) |
| Parkinson’s Disease | 893 (0.2) | 945 (0.2) |
| Migraine | 53,130 (1.3) | 52,587 (1.1) |
| Multiple Sclerosis | 1,032 (0.2) | 1,008 (0.2) |
| Epilepsy | 7,495 (1.6) | 7,505 (1.6) |
| Hemiplegia | 710 (0.2) | 703 (0.1) |
| Chronic Fatigue Syndrome | 1,670 (0.4) | 1,667 (0.4) |
| Fibromyalgia | 5,115 (1.1) | 5,027 (1.1) |
| Cluster Headache | 1,758 (0.4) | 1,685 (0.4) |
| Osteoarthritis | 52,342 (11.1) | 52,316 (11.1) |
| Backpain | 6,835 (1.4) | 6,826 (1.4) |
| Fragility Fracture | 45,198 (9.6) | 44,943 (9.5) |
| Falls | 37,672 (8.0) | 38,146 (8.1) |
| Polymyalgia Rheumatica | 2,077 (0.4) | 2,088 (0.4) |
| Rheumatoid Arthritis | 3,991 (0.8) | 4,043 (0.9) |
| Raynaud’s Disease | 5,211 (1.1) | 5,218 (1.1) |
| Sjogren’s Syndrome | 539 (0.1) | 532 (0.1) |
| Systemic Lupus Erythematosus | 600 (0.1) | 592 (0.1) |
| Systemic Sclerosis | 148 (0.0) | 156 (0.0) |
| Ankylosing Spondylitis | 777 (0.2) | 764 (0.2) |

**Supplementary Table 10.** Comorbidities at baseline in matched exposed and unexposed groups **(Continued)**

| **Comorbidity** | **Unexposed** | **Exposed** |
| --- | --- | --- |
| Gout | 13,845 (2.9) | 13,703 (2.9) |
| Chronic Kidney Disease | 13,630 (2.9) | 13,866 (2.9) |
| Asthma | 95,329 (20.2) | 94,787 (20.1) |
| Chronic Obstructive Pulmonary Disease | 10,494 (2.2) | 10,612 (2.2) |
| Obstructive Sleep Apnoea | 7,054 (1.5) | 6,871 (1.5) |
| Other Pulmonary Disease | 2,604 (0.6) | 2,547 (0.5) |
| Hyperthyroidism | 5,226 (1.1) | 5,126 (1.1) |
| Hypothyroidism | 20,720 (4.4) | 20,565 (4.4) |
| Type 1 Diabetes | 2,760 (0.6) | 2,752 (0.6) |
| Type 2 Diabetes | 31,899 (6.8) | 31,673 (6.7) |
| Acquired Immune Deficiency Syndrome | 897 (0.2) | 895 (0.2) |
| Benign Prostatic Hyperplasia | 6,551 (1.4) | 6,574 (1.4) |
| Erectile Dysfunction | 21,407 (4.5) | 20,862 (4.4) |

### **Supplementary Table 11.** Incremental cost OLS regression estimates for primary care healthcare cost associated with Long COVID

| **Total healthcare cost** | **Coefficient** | **95% CIs (LL, UL)** | | **p-value** |
| --- | --- | --- | --- | --- |
| Exposure status |  |  |  |  |
| Unexposed (Reference) |  |  |  |  |
| Covid-19 | 2.09 | 1.95 | 2.24 | <0.001 |
| Long Covid diagnosed | 20.50 | 18.50 | 22.50 | <0.001 |
| Symptoms of long Covid | 39.61 | 38.94 | 40.28 | <0.001 |
| Charlson Comorbidity Index | 0.49 | 0.43 | 0.55 | <0.001 |
| GP consultations prior | 2.50 | 2.43 | 2.57 | <0.001 |
| Nurse consultations prior | 1.24 | 1.09 | 1.39 | <0.001 |
| Physio consultations prior | 1.85 | 1.14 | 2.56 | <0.001 |
| Age (at index) | 0.15 | 0.14 | 0.15 | <0.001 |
| Time since index date  Sex |  | 1.81 | 1.85 | <0.001 |
| Male (Reference) |  |  |  |  |
| Female | 1.54 | 1.39 | 1.70 | <0.001 |
| Ethnicity |  |  |  |  |
| White (Reference) |  |  |  |  |
| Black | -0.60 | -0.94 | -0.26 | <0.001 |
| Other | 0.01 | -0.53 | 0.55 | 0.97 |
| Mixed | -0.02 | -0.23 | 0.20 | 0.87 |
| Asian | -0.59 | -1.01 | -0.16 | 0.01 |
| Ethnicity missing | -0.26 | -0.46 | -0.06 | 0.01 |
| IMD |  |  |  |  |
| 1 (Least deprived) (Reference) |  |  |  |  |
| 2 | 0.36 | 0.10 | 0.62 | 0.01 |
| 3 | 0.67 | 0.41 | 0.92 | <0.001 |
| 4 | 0.53 | 0.28 | 0.77 | <0.001 |
| 5 (Most deprived) | 0.82 | 0.56 | 1.07 | <0.001 |
| IMD missing  Smoking status | 0.04 | -0.27 | 0.34 | 0.82 |
| Non-smoker (Reference) |  |  |  |  |
| Ex-smoker | 0.30 | 0.12 | 0.49 | <0.001 |
| Current smoker | 0.14 | -0.05 | 0.33 | 0.14 |
| Smoking status missing | 0.23 | -0.01 | 0.47 | 0.06 |
| BMI category: |  |  |  |  |
| Normal weight (Reference) |  |  |  |  |
| Underweight | 1.48 | 1.08 | 1.88 | <0.001 |
| Overweight | -0.41 | -0.60 | -0.23 | <0.001 |
| Obese | 0.21 | -0.00 | 0.43 | 0.05 |
| BMI missing | 0.65 | 0.41 | 0.89 | <0.001 |
| Constant | -35.27 | -36.07 | -34.47 | <0.001 |

*Notes: R^2^ = 0.297, MSE = 35.642*

### **Supplementary Table 12.** Baseline characteristics for the COVID patients used in the part 2 regression analysis (incurred a positive cost)

| **Variables** | **COVID-19 patients**  **(n = 98,476)** |
| --- | --- |
| Age at index (mean (SD)) | 46.85 (18.10) |
| Gender |  |
| Male | 33,142 (33.7) |
| Female | 65,334 (66.3) |
| BMI category |  |
| Normal weight | 29,383 (29.8) |
| Underweight | 3,052 (3.1) |
| Obese | 29,356 (29.8) |
| Overweight | 30,779 (31.3) |
| Missing | 5,906 (6.0) |
| IMD |  |
| 1 (Least deprived) | 14,839 (15.1) |
| 2 | 15,737 (16.0) |
| 3 | 16,422 (16.7) |
| 4 | 19,702 (20.0) |
| 5 (Most deprived) | 22,617 (23.0) |
| Missing | 9,159 (9.3) |
| Smoking Status |  |
| Current smoker | 20,710 (21.0) |
| Ex-Smoker | 38,929 (39.5) |
| Never smoked | 30,862 (31.3) |
| Missing | 7,975 (8.1) |
| Ethnicity |  |
| White | 66,078 (67.1) |
| Asian | 12,915 (13.1) |
| Black | 3,177 (3.2) |
| Mixed | 1,812 (1.8) |
| Other | 1,239 (1.3) |
| Missing | 13,255 (13.5) |
| Number of consultations 3 to 12 months prior to index date (mean (SD)) |  |
| GP | 3.64 (4.53) |
| Nurse | 0.86 (2.05) |
| Physiotherapist | 0.02 (0.24) |
| Surgery | 2.21 (2.83) |
| Home visits | 0.18 (1.22) |
| Telephone | 2.09 (3.20) |
| Triage | 0.04 (0.37) |

**Supplementary Table 12.** Baseline characteristics for the COVID patients used in the part 2 regression analysis (incurred a positive cost) **(Continued)**

| **Variables** | **COVID-19 patients**  **(n = 98,476)** |
| --- | --- |
| Cancer | 5,941 (6.0) |
| Arrhythmia | 10,437 (10.6) |
| Atrial Fibrillation | 3,100 (3.1) |
| Hypertension | 20,091 (20.4) |
| Heart Failure | 1,457 (1.5) |
| Ischaemic Heart Disease | 4,313 (4.4) |
| Myocardial Infarction | 2,056 (2.1) |
| Valvular Heart Disease | 1,851 (1.9) |
| Cardiomyopathy | 345 (0.4) |
| Congenital Heart Disease | 687 (0.7) |
| Peripheral Vascular Disease | 1,338 (1.4) |
| Aortic Aneurysm | 294 (0.3) |
| Transient Ischaemic Attack | 1,453 (1.5) |
| Ischaemic Stroke | 758 (0.8) |
| Haemorrhagic Stroke | 381 (0.4) |
| Stroke Unspecified | 1,199 (1.2) |
| Eczema | 21,821 (22.2) |
| Psoriasis | 4,823 (4.9) |
| Autoimmune Skin Conditions | 1,446 (1.5) |
| Acne | 14,751 (15.0) |
| Hay Fever | 20,430 (20.7) |
| Chronic Sinusitis | 2,515 (2.6) |
| Deafness | 1,564 (1.6) |
| Blindness | 672 (0.7) |
| Cataract | 5,200 (5.3) |
| Glaucoma | 1,459 (1.5) |
| Age-related Macular Degeneration | 1,121 (1.1) |
| Diabetic Retinopathy | 3,972 (4.0) |
| Inflammatory Eye Disease | 2,150 (2.2) |
| Peptic Ulcer | 1,978 (2.0) |
| Inflammatory Bowel Disease | 1,336 (1.4) |
| Irritable Bowel Syndrome | 10,217 (10.4) |
| Hepatitis B | 258 (0.3) |
| Hepatitis C | 216 (0.2) |
| Alcohol Related Chronic Liver Disease | 251 (0.3) |
| Chronic Liver Disease (All) | 3,961 (4.0) |
| Non-Alcoholic Fatty Liver Disease | 1,835 (1.9) |
| Diverticular Disease | 4,497 (4.6) |
| Coeliac Disease | 588 (0.6) |
| Chronic Pancreatitis | 121 (0.1) |
| Endometriosis | 2,158 (2.2) |

**Supplementary Table 12.** Baseline characteristics for the COVID patients used in the part 2 regression analysis (incurred a positive cost) **(Continued)**

| **Variables** | **COVID-19 patients**  **(n = 98,476)** |
| --- | --- |
| Poly Cystic Ovarian Syndrome | 3,283 (3.3) |
| Low Haemoglobin | 7,982 (8.1) |
| Venous Thromboembolism | 2,916 (3.0) |
| Coagulopathy | 1,511 (1.5) |
| Pernicious Anaemia | 464 (0.5) |
| Depression | 29,482 (29.9) |
| Anxiety | 27,422 (27.8) |
| Serious Mental Illness | 1,682 (1.7) |
| Substance Misuse | 2,073 (2.1) |
| Alcohol Misuse | 5,898 (6.0) |
| Attention Deficit Hyperactivity Disorder | 505 (0.5) |
| Eating Disorder | 1,311 (1.3) |
| Learning Disability | 1,092 (1.1) |
| Alzheimer’s | 1,213 (1.2) |
| Vascular Dementia | 665 (0.7) |
| Dementia Unspecified | 2,613 (2.7) |
| Parkinson’s Disease | 300 (0.3) |
| Migraine | 14,707 (14.9) |
| Multiple Sclerosis | 310 (0.3) |
| Epilepsy | 1,942 (2.0) |
| Hemiplegia | 204 (0.2) |
| Chronic Fatigue Syndrome | 567 (0.6) |
| Fibromyalgia | 1,923 (2.0) |
| Cluster Headache | 468 (0.5) |
| Osteoarthritis | 16,242 (16.5) |
| Backpain | 2140 (2.2) |
| Fragility Fracture | 10,550 (10.7) |
| Falls | 11,472 (11.6) |
| Polymyalgia Rheumatica | 762 (0.8) |
| Rheumatoid Arthritis | 1,320 (1.3) |
| Raynaud’s Disease | 1,462 (1.5) |
| Sjogren’s Syndrome | 196 (0.2) |
| Systemic Lupus Erythematosus | 172 (0.2) |
| Systemic Sclerosis | 47 (0.0) |
| Ankylosing Spondylitis | 205 (0.2) |
| Gout | 3,668 (3.7) |
| Chronic Kidney Disease | 4,612 (4.7) |
| Asthma | 23,792 (24.2) |
| Chronic Obstructive Pulmonary Disease | 3,577 (3.6) |
| Obstructive Sleep Apnoea | 1,992 (2.0) |
| Other Pulmonary Disease | 880 (0.9) |

**Supplementary Table 12.** Baseline characteristics for the COVID patients used in the part 2 regression analysis (incurred a positive cost) **(Continued)**

| **Variables** | **COVID-19 patients**  **(n = 98,476)** |
| --- | --- |
| Hyperthyroidism | 1,508 (1.5) |
| Hypothyroidism | 6,282 (6.4) |
| Type 1 Diabetes | 743 (0.8) |
| Type 2 Diabetes | 9,714 (9.9) |
| Acquired Immune Deficiency Syndrome | 204 (0.2) |
| Benign Prostatic Hyperplasia | 1,968 (2.0) |
| Erectile Dysfunction | 5,371 (5.5) |

### **Supplementary Table 13.** The mean cost of healthcare professional consultations for COVID-19 patients, stratified by subgroups of patients

|  | **Healthcare professionals – Mean (SD)** | | |  |  |
| --- | --- | --- | --- | --- | --- |
| **Characteristics** | **GP** | **Nurse** | **Physio** | **All** | **p-value *** |
| Age-group |  |  |  |  | < 0.001 |
| 18-29 | 27.87 (3.47) | 11.74 (5.79) | 36.16 (33.15) | 54.23 (51.46) |  |
| 30-39 | 27.96 (3.54) | 11.7 (5.99) | 32.13 (33.74) | 57.13 (60.09) |  |
| 40-49 | 28.03 (3.58) | 11.62 (6.02) | 30.14 (32.91) | 60.42 (64.79) |  |
| 50-59 | 28.09 (3.62) | 11.42 (5.96) | 35.8 (33.48) | 63.46 (70.39) |  |
| 60-69 | 28.22 (3.92) | 11.53 (6.09) | 38.33 (32.58) | 68.04 (80.81) |  |
| 70-79 | 28.89 (4.78) | 12.2 (7.21) | 30.33 (33.04) | 89.08 (120.21) |  |
| 80s and over | 29.71 (5.8) | 13.14 (7.92) | 15.75 (29.8) | 129.74 (150.24) |  |
| Sex |  |  |  |  | < 0.001 |
| Male | 28.34 (4.1) | 11.66 (6.38) | 32.86 (33.29) | 64.44 (78.45) |  |
| Female | 28.24 (4.02) | 11.87 (6.28) | 33.28 (33.5) | 66.98 (79.81) |  |
| Ethnicity |  |  |  |  | < 0.001 |
| White | 28.29 (4.09) | 11.78 (6.33) | 33.41 (33.5) | 67.26 (82.26) |  |
| Asian | 28.15 (3.64) | 12.11 (6.51) | 31.92 (33.29) | 63.56 (65.92) |  |
| Black | 28.15 (3.76) | 11.51 (5.92) | 23.56 (31.84) | 64.04 (73.28) |  |
| Mixed | 28.01 (3.63) | 11.96 (6.06) | 27.58 (32.62) | 62.13 (62.82) |  |
| Other | 28.17 (3.88) | 12.15 (5.73) | 32.5 (32.81) | 63.92 (86.41) |  |
| Missing | 28.41 (4.32) | 11.73 (6.18) | 35.02 (32.94) | 64.21 (79.32) |  |

**Supplementary Table 13.** The mean cost of healthcare professional consultations for COVID-19 patients, stratified by subgroups of patients **(Continued)**

| IMD |  |  |  |  | < 0.001 |
| --- | --- | --- | --- | --- | --- |
| 1 (Least deprived) | 28.25 (4.15) | 11.49 (6.02) | 32.28 (33.24) | 64.01 (80.65) |  |
| 2 | 28.37 (4.19) | 11.55 (6.05) | 30.69 (33.18) | 66.44 (84.72) |  |
| 3 | 28.29 (4.07) | 11.71 (6.14) | 30.93 (32.68) | 68.19 (84.13) |  |
| 4 | 28.22 (3.92) | 11.9 (6.51) | 37.1 (33.03) | 64.99 (76.81) |  |
| 5 (Most deprived) | 28.25 (3.99) | 12.11 (6.44) | 32.51 (34.58) | 67.09 (75.8) |  |
| Missing | 28.28 (3.96) | 11.88 (6.66) | 34.72 (32.8) | 65.39 (72.63) |  |
| Smoking Status |  |  |  |  | < 0.001 |
| Current smoker | 28.2 (3.94) | 11.95 (6.59) | 28.59 (33.17) | 64.48 (75.48) |  |
| Ex-smoker | 28.34 (4.13) | 11.68 (6.29) | 33.74 (33.51) | 70.11 (86.97) |  |
| Never smoked | 28.25 (4) | 11.87 (6.16) | 35.74 (33.05) | 63.47 (73.98) |  |
| Missing | 28.24 (4) | 11.94 (6.14) | 31.72 (33.14) | 61.23 (68.92) |  |
| BMI categories |  |  |  |  | < 0.001 |
| Obese | 28.18 (3.86) | 11.6 (6.14) | 32.78 (33.35) | 70.73 (86.19) |  |
| Overweight | 28.27 (4.02) | 11.78 (6.4) | 32.84 (33.71) | 64.97 (75.88) |  |
| Under/Normal weight | 28.33 (4.14) | 12 (6.38) | 33.92 (32.98) | 63.69 (74.88) |  |
| Underweight | 28.55 (4.63) | 11.87 (6.52) | 28.98 (34.74) | 67.68 (86.23) |  |
| Missing | 28.48 (4.31) | 12.51 (6.4) | 32.85 (33.1) | 59.19 (75.94) |  |
| Region |  |  |  |  | < 0.001 |
| Northeast | 28.68 (4.46) | 10.96 (4.61) | 39.87 (43.01) | 68.68 (73.86) |  |
| Northwest | 28.33 (4.02) | 12.15 (6.56) | 30.49 (32.68) | 70.21 (81.57) |  |
| Yorkshire and the Humber | 27.77 (4.05) | 10.86 (5.21) | 32.67 (35.53) | 69.76 (85.74) |  |
| East Midlands | 27.66 (3.41) | 13.54 (8.36) | 37.49 (33.82) | 67.75 (69.93) |  |
| West Midlands | 28.18 (3.91) | 11.91 (6.57) | 35.24 (32.9) | 66.46 (77.21) |  |
| East of England | 28.11 (4.35) | 12.41 (5.74) | 36.9 (33.45) | 64.34 (75.01) |  |
| Southwest | 28.31 (3.96) | 11.72 (6.89) | 23.05 (31.25) | 66.17 (82.13) |  |
| South Central | 28.34 (4.37) | 10.81 (5.55) | 37.05 (32.85) | 67.41 (88.05) |  |
| London | 28.28 (3.98) | 11.53 (5.99) | 32.33 (33.09) | 60.24 (75.24) |  |
| Southeast Coast | 28.51 (4.06) | 11.53 (5.3) | 39.73 (31.87) | 59.67 (78.74) |  |
| Northern Ireland | 27.49 (5.97) | 11.22 (0.24) | N/A | 77.43 (64.24) |  |

*(*Bootstrapped t-test and ANOVA)*

### **Supplementary Table 14.** Estimates of first three months (after index date + 12 weeks) of primary care resource use and costs associated with Long COVID, for patients who had at least three months of follow-up. (Sensitivity analysis)

|  | **Main analysis groups:** | | **COVID-19 patients with:** | | |
| --- | --- | --- | --- | --- | --- |
| **Cost component** | **Unexposed (n = 64,604)** | **Exposed (n = 66,303)** | **DLC  (n = 444)** | | **SLC (n = 10,310)** |
| **Consultations 12 weeks after index date** | | | | | |
| Count | 60,817 | 77,222 | 1,558 | 29,319 | |
| Rate | 0.94 | 1.16^***^ | 3.51^***^ | 2.84^***^ | |
| **Cost (absolute)** | | | | | |
| Total | £1,545,629 | £1,965,131 | £41,059 | £730,980 | |
| Per patient | £23.92 | £29.64^***^ | £92.47^***^ | £70.90^***^ | |
| Per patient who had at least one consultation | £65.34 | £69.52^***^ | £108.62^***^ | £90.24^***^ | |
| **Cost (per person year)** | | | | | |
| Total | £2,507,819 | £3,224,856 | £68,601 | £1,155,146 | |
| Mean | £38.82 | £48.64^***^ | £154.51^***^ | £112.04^***^ | |
| Mean (patients who had at least one consultation) | £105.38 | £113.51^***^ | £180.87^***^ | £142.42^***^ | |

*Notes: DLC – Diagnosed with Long COVID patient group. SLC – Symptoms of Long COVID patient group. Key: ^*^p<0.05, ^**^p<0.01, ^***^ p<0.001*
